# Supplementary figures and images for: DHODH inhibition enhances the efficacy of immune checkpoint blockade by increasing cancer cell antigen presentation
Source: bioRxiv. 2024 Jan 13:2023.04.03.535399. Originally published 2023 Apr 5. Preprint. [Version 2] doi: 10.1101/2023.04.03.535399 (PMC10103971; doi:10.1101/2023.04.03.535399)

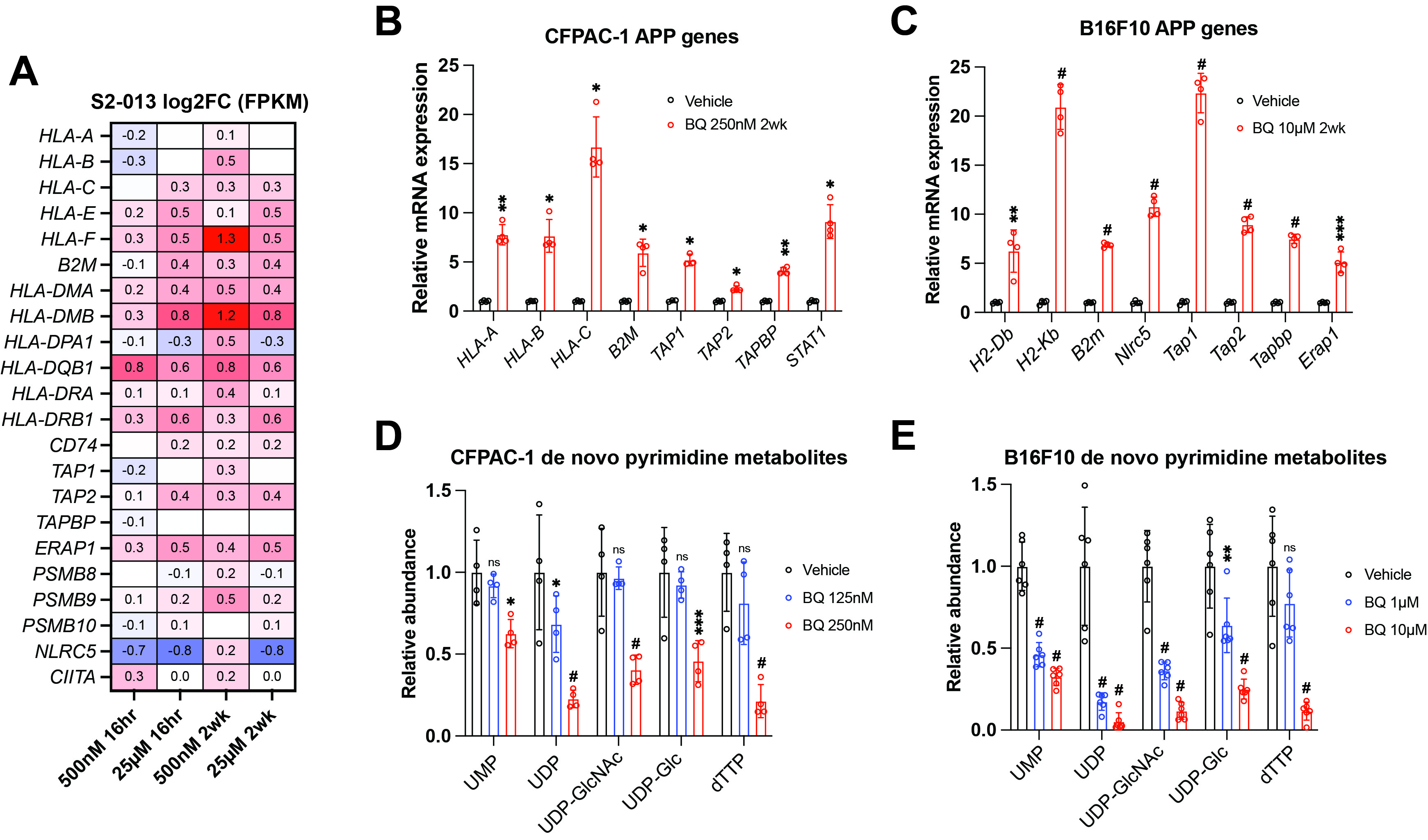

Supplement: Supplement 1 — Figure S1: BQ treatment upregulates APP genes and depletes pyrimidine nucleotides. A) Heatmap showing log2 fold change mRNA expression of APP genes in S2–013 cells treated with BQ for indicated dose and duration. B-C) RT-qPCR quantification of APP genes after two-week BQ treatment of CFPAC-1 (250nM) (B) or B16F10 (10μM) (C) cells. D-E) Quantification of pyrimidine metabolites in CFPAC-1 (C) or B16F10 (D) cells treated with BQ for 8 hours at indicated doses. Data represent mean +/− SEM of four (CFPAC-1) or six (B16F10) biological replicates. * indicates p < 0.05, ** p < 0.01, *** p < 0.001, and # p < 0.0001 by unpaired t-test (B, C) or two-way ANOVA with Bonferonni’s post-comparison test (D, E). [file media-1.jpg]

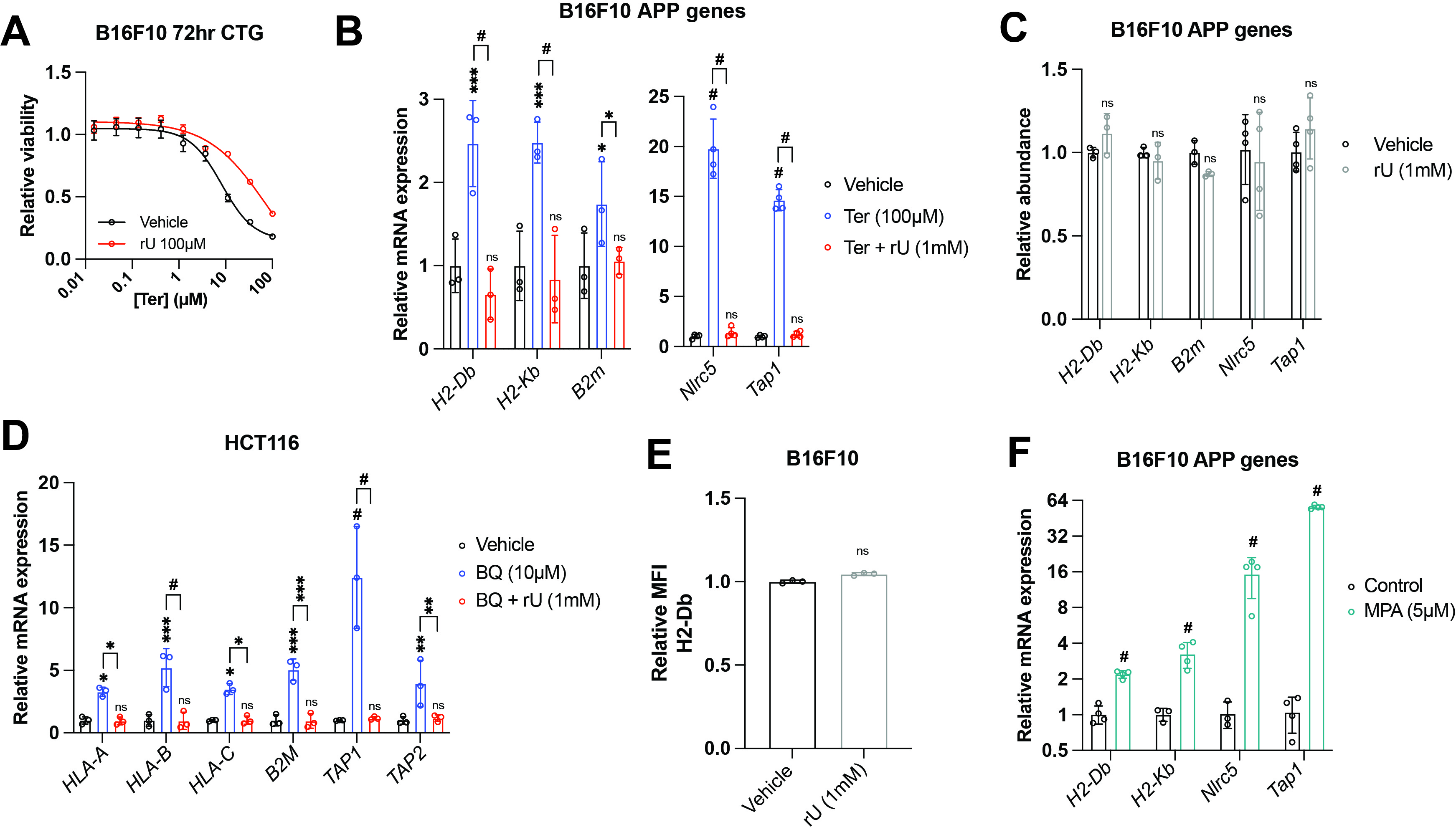

Supplement: Supplement 2 — Figure S2: Uridine rescues B16F10 cells from teriflunomide toxicity but does not alter APP expression by itself. A) Dose-response cell viability experiment as in Fig 2A but with teriflunomide (Ter) instead of BQ. B) RT-qPCR analysis of indicated genes following treatment with teriflunomide +/− uridine (1mM) for 24 hours. Data represent mean +/− SEM of three determinations. One representative result of three independent experiments is shown. C) RT-qPCR analysis of indicated genes after treatment with vehicle or uridine (1mM) for 24 hours. Data represent mean +/− SEM of four determinations. One representative result of three independent experiments is shown. D) RT-qPCR analysis of indicated genes following treatment with BQ (10μM) +/− uridine (1mM) for 24 hours. Data represent mean +/− SD of three independent experiments. E) Flow cytometry analysis of cell surface MHC-I (H2-Db) following 24-hour uridine (1mM) treatment. Data represent mean +/− SEM of three independent experiments. F) RT-qPCR analysis of indicated genes in B16F10 cells following 24-hour treatment with MPA (5μM). Data represent mean +/− SEM of four determinations. One representative result of three independent experiments is shown. [file media-2.jpg]

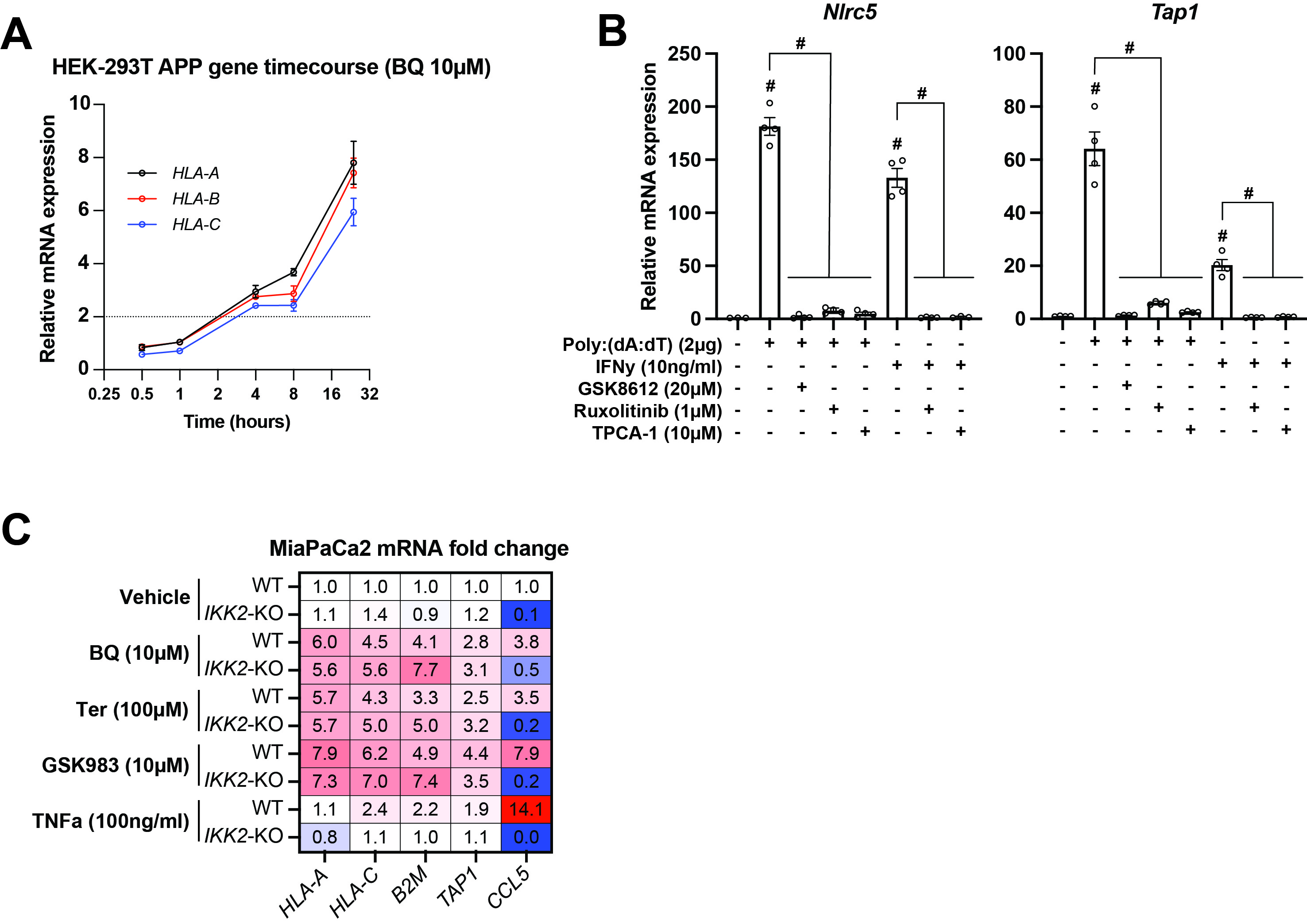

Supplement: Supplement 3 — Figure S3: A) RT-qPCR time course analysis for MHC-I genes in HEK-293T cells treated for indicated times with BQ (10μM). Data represent mean +/− SD of four determinations. B) RT-qPCR analysis for Nlrc5 (left) or Tap1 (right) in B16F10 cells treated for 24 hours with indicated agents. Data represent mean +/− SD of four determinations. # indicates p < 0.0001 with two-way ANOVA with Bonferroni post-comparison test. Representative results for one of three independent experiments are shown. C) Heatmap indicating RT-qPCR analysis for indicated genes in wild-type or IKK2-KO MiaPaCa2 cells treated with indicated agents for 24 hours. Numbers in the heatmap represent mean of four determinations. [file media-3.jpg]

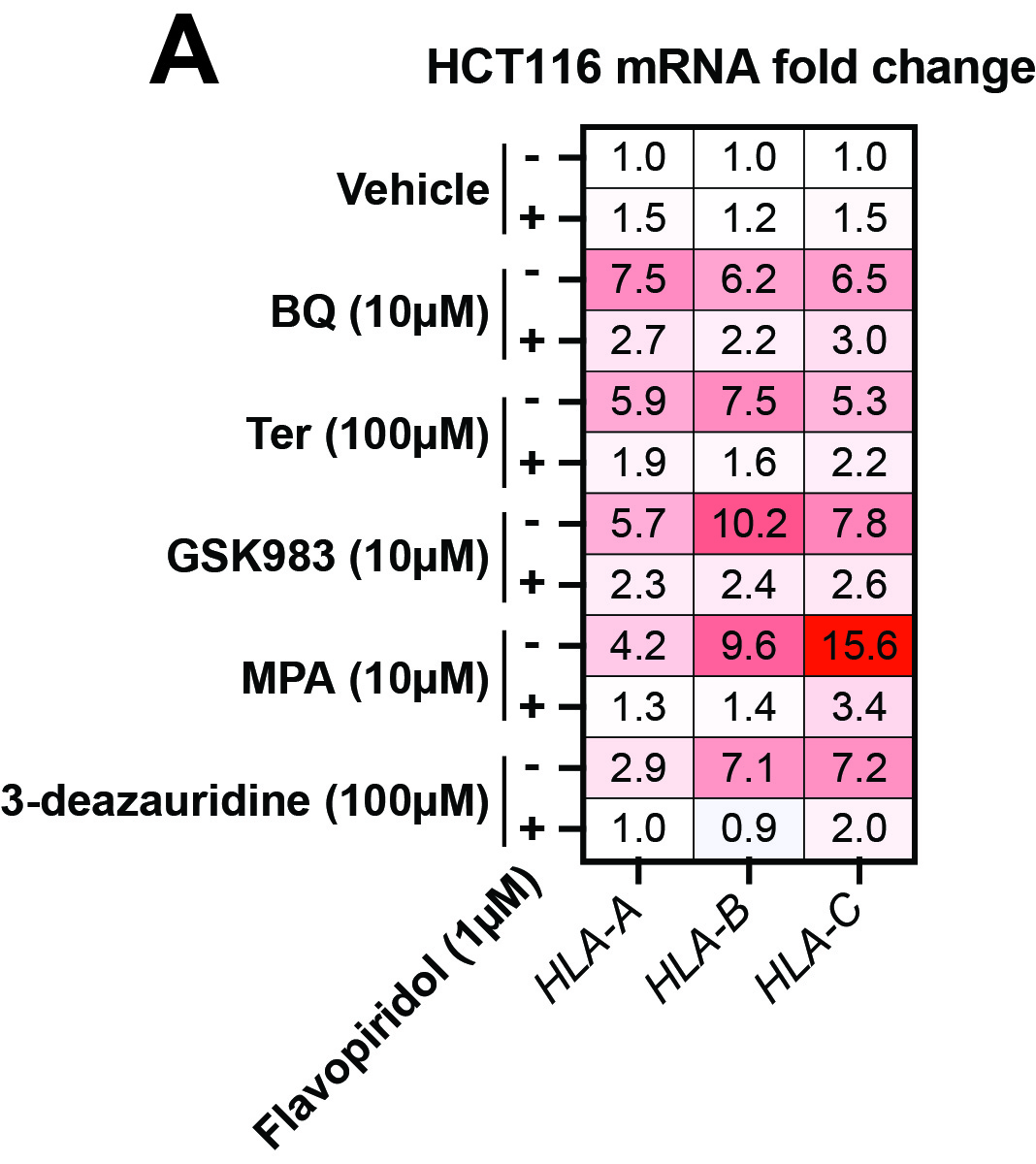

Supplement: Supplement 4 — Figure S4: A) RT-qPCR analysis for indicated genes in HCT116 cells treated with indicated agents in the presence or absence of flavopiridol (1μM). Numbers in the heatmap represent mean of three determinations. [file media-4.jpg]

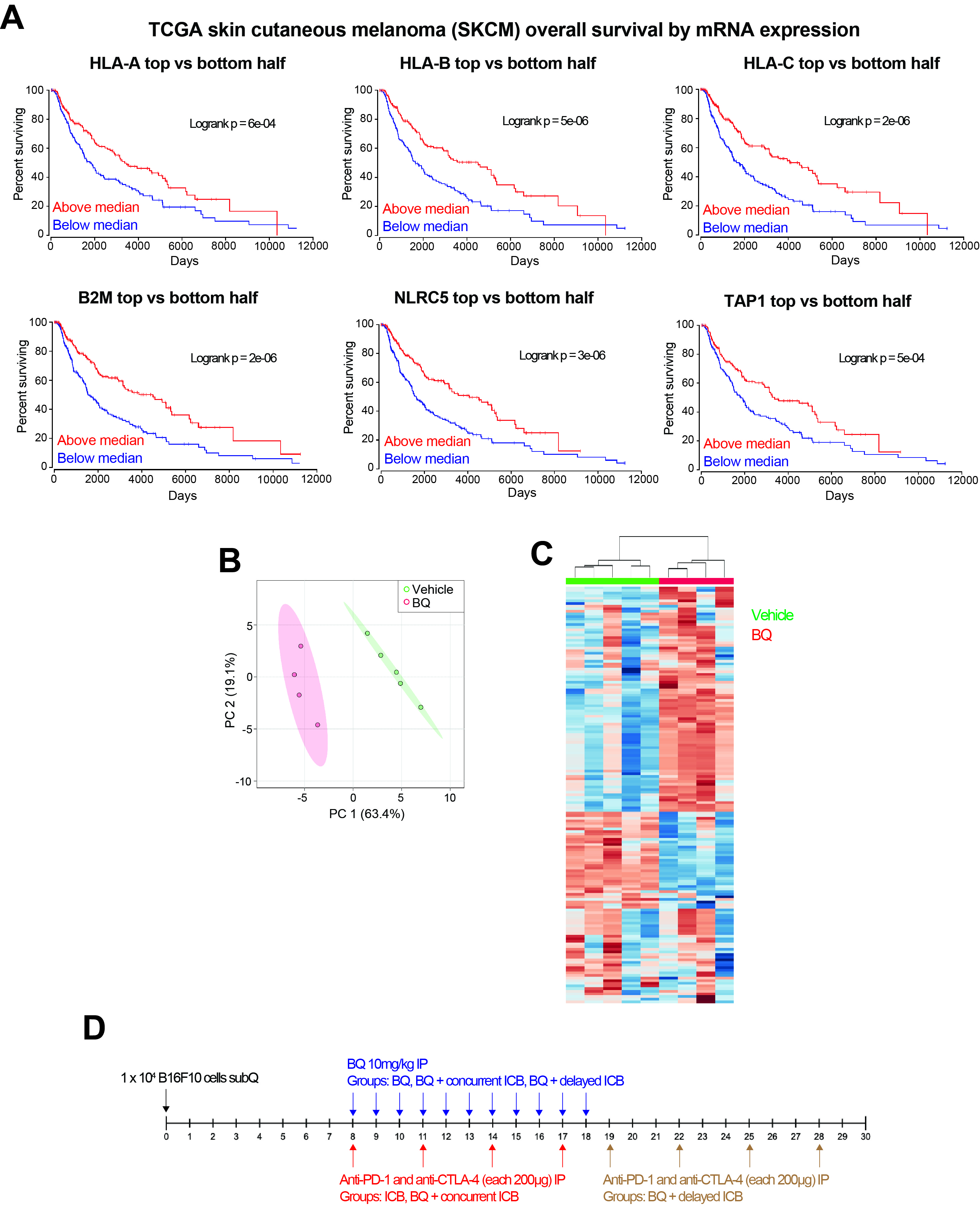

Supplement: Supplement 5 — Figure S5: A) Overall survival in melanoma patients (SKCM) from the Cancer Genome Atlas (TCGA) with above (indicated in red) and below median (indicated in blue) mRNA expression of indicated genes. B-C) Principal component analysis (PCA) plot (B) and unsupervised hierarchical clustering (C) from metabolomics analysis of B16F10 tumors at necropsy (Fig 5C). D) Treatment regimen of mice from Fig 5E. [file media-5.jpg]
