# Supplementary material for: DHODH inhibition enhances the efficacy of immune checkpoint blockade by increasing cancer cell antigen presentation
Source: bioRxiv. 2023 Apr 5:2023.04.03.535399. Preprint. [Version 1] doi: 10.1101/2023.04.03.535399 (PMC10103971; doi:10.1101/2023.04.03.535399)
Supplement: Supplementary file 1 [file NIHPP2023.04.03.535399v1-supplement-1.pdf]

bioRxiv preprint doi: https://doi.org/10.1101/2023.04.03.535399; this version posted April 5, 2023. The copyright holder for this preprint (which was not certified by peer review) is the author/funder, who has granted bioRxiv a license to display the preprint in perpetuity. It is made available under aCC-BY 4.0 International license.

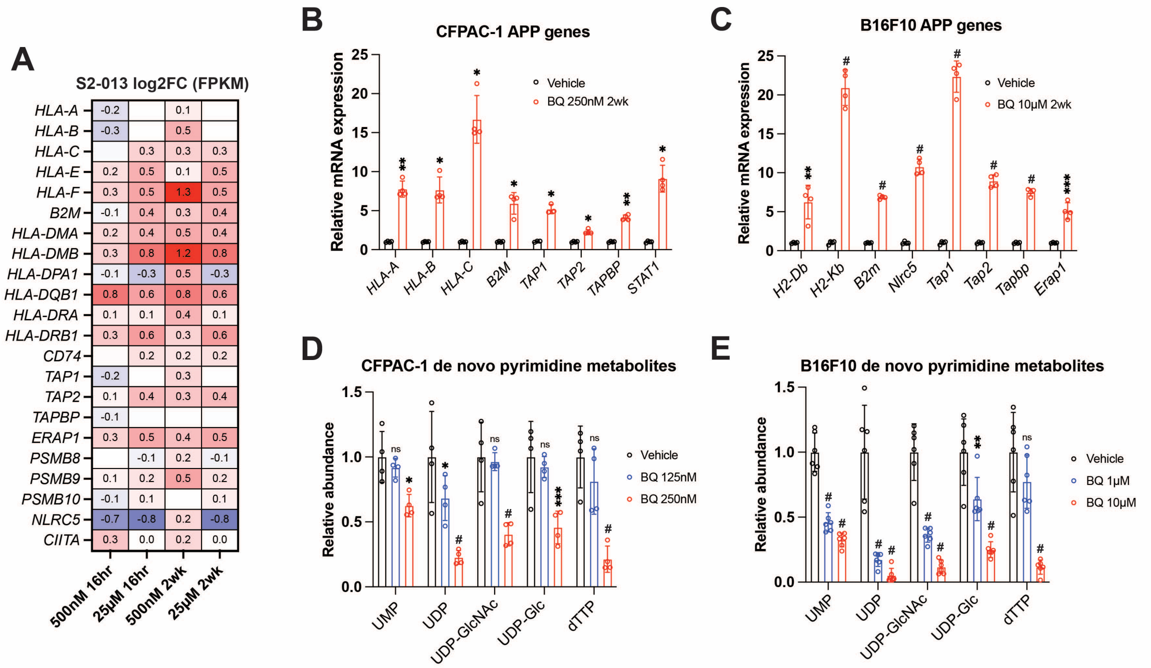

Figure S1: BQ treatment upregulates APP genes and depletes pyrimidine nucleotides. A) Heatmap showing log2 fold change mRNA expression of APP genes in S2-013 cells treated with BQ for indicated dose and duration. **B-C)** RT-qPCR quantification of APP genes after two-week BQ treatment of CFPAC-1 (250nM) (B) or B16F10 (10µM) (C) cells. **D-E)** Quantification of pyrimidine metabolites in CFPAC-1 (C) or B16F10 (D) cells treated with BQ for 8 hours at indicated doses. Data represent mean +/- SEM of four (CFPAC-1) or six (B16F10) biological replicates. * indicates $p < 0.05$, ** $p < 0.01$, *** $p < 0.001$, and # $p < 0.0001$ by unpaired t-test (B, C) or two-way ANOVA with Bonferonni's post-comparison test (D, E).

bioRxiv preprint doi: https://doi.org/10.1101/2023.04.03.535399; this version posted April 5, 2023. The copyright holder for this preprint (which was not certified by peer review) is the author/funder, who has granted bioRxiv a license to display the preprint in perpetuity. It is made available under aCC-BY 4.0 International license.

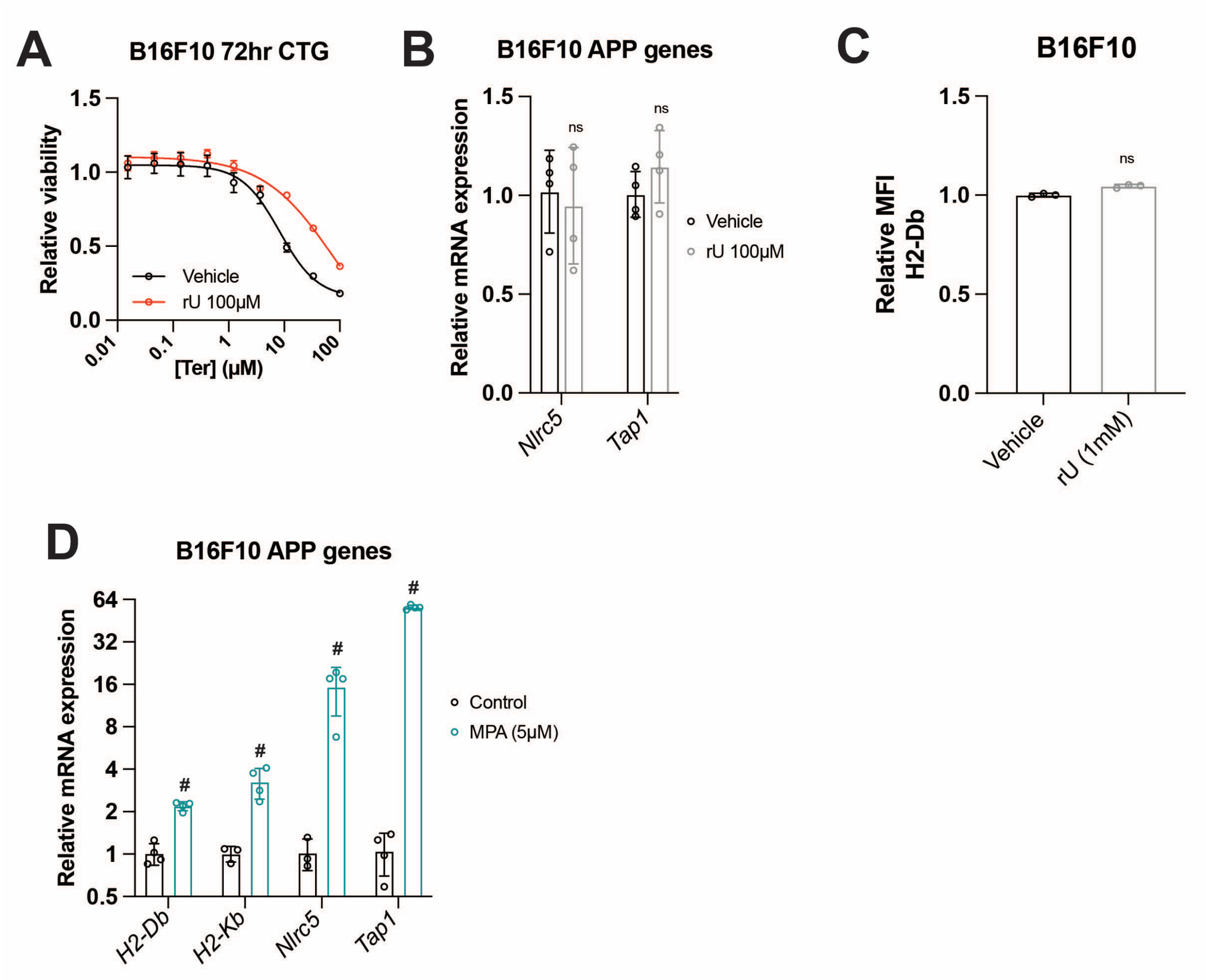

Figure S2: Uridine rescues B16F10 cells from teriflunomide toxicity but does not alter APP expression by itself. A) Dose-response cell viability experiment as in Fig 2A but with teriflunomide (Ter) instead of BQ. **B)** RT-qPCR analysis of indicated genes +/- single agent uridine treatment for 24 hours. Data represent mean +/- SEM of 4 determinations. One representative result of three independent experiments is shown. **C)** Flow cytometry analysis of cell surface MHC-I (H2-Db) following 24-hour uridine (1mM) treatment. Data represent mean +/- SEM of three independent experiments. **D)** RT-qPCR analysis of indicated genes in B16F10 cells following 24-hour treatment with MPA (5μM). Data represent mean +/- SEM of four determinations. One representative result of three independent experiments is shown.

bioRxiv preprint doi: https://doi.org/10.1101/2023.04.03.535399; this version posted April 5, 2023. The copyright holder for this preprint (which was not certified by peer review) is the author/funder, who has granted bioRxiv a license to display the preprint in perpetuity. It is made available under aCC-BY 4.0 International license.

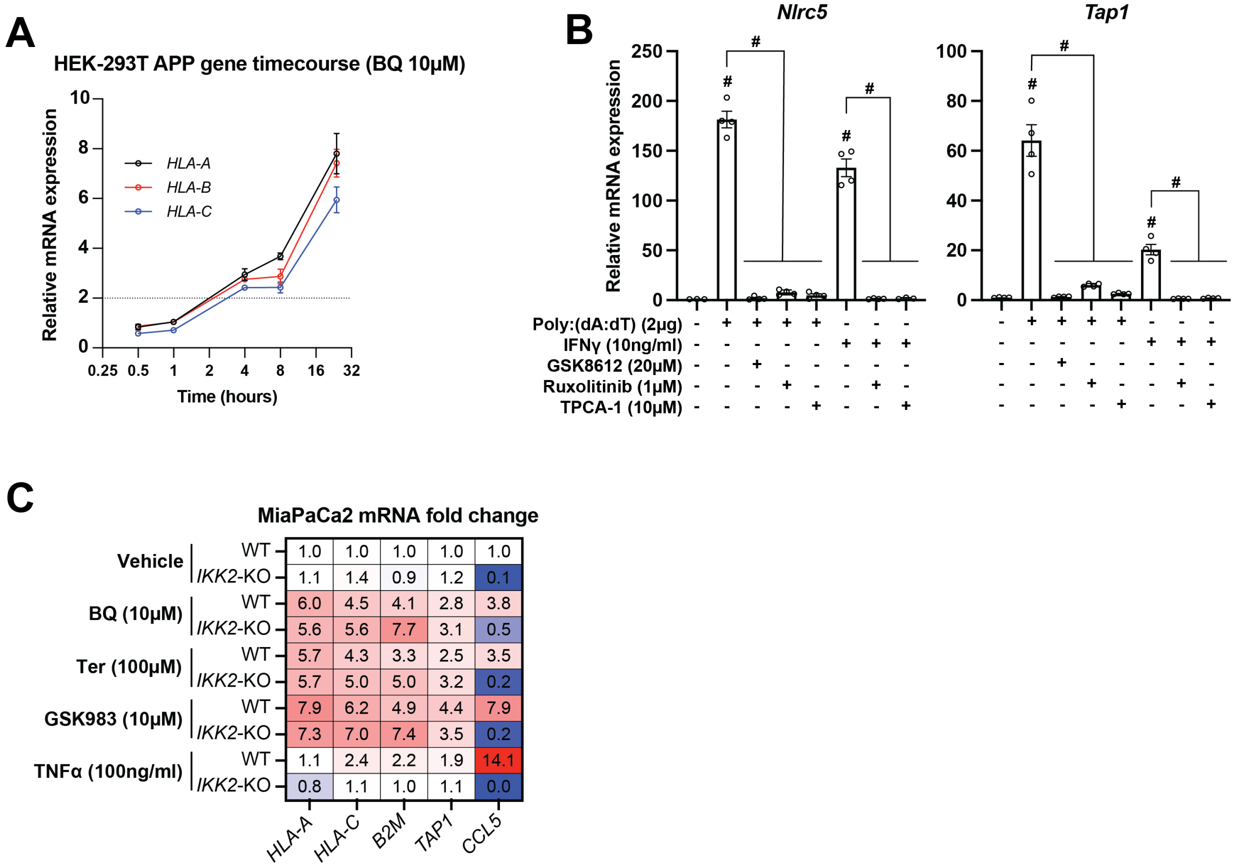

Figure S3: A) RT-qPCR time course analysis for MHC-I genes in HEK-293T cells treated for indicated times with BQ (10μM). Data represent mean +/- SD of four determinations. **B)** RT-qPCR analysis for *Nlrc5* (left) or *Tap1* (right) in B16F10 cells treated for 24 hours with indicated agents. Data represent mean +/- SD of four determinations. # indicates p < 0.0001 with two-way ANOVA with Bonferroni post-comparison test. Representative results for one of three independent experiments are shown. **C)** Heatmap indicating RT-qPCR analysis for indicated genes in wild-type or *IKK2*-KO MiaPaCa2 cells treated with indicated agents for 24 hours. Numbers in the heatmap represent mean of four determinations.

bioRxiv preprint doi: https://doi.org/10.1101/2023.04.03.535399; this version posted April 5, 2023. The copyright holder for this preprint (which was not certified by peer review) is the author/funder, who has granted bioRxiv a license to display the preprint in perpetuity. It is made available under aCC-BY 4.0 International license.

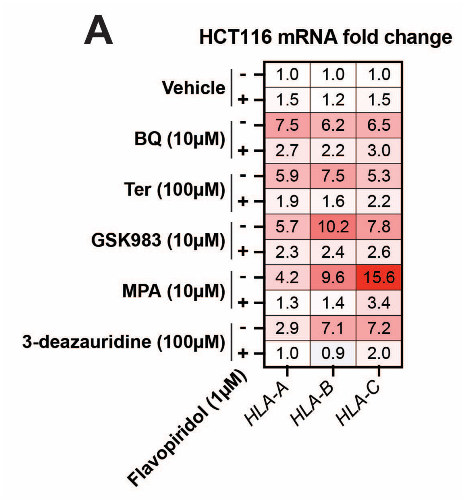

Figure S4: A) RT-qPCR analysis for indicated genes in HCT116 cells treated with indicated agents in the presence or absence of flavopiridol (1μM). Numbers in the heatmap represent mean of three determinations.

bioRxiv preprint doi: https://doi.org/10.1101/2023.04.03.535399; this version posted April 5, 2023. The copyright holder for this preprint (which was not certified by peer review) is the author/funder, who has granted bioRxiv a license to display the preprint in perpetuity. It is made available under aCC-BY 4.0 International license.

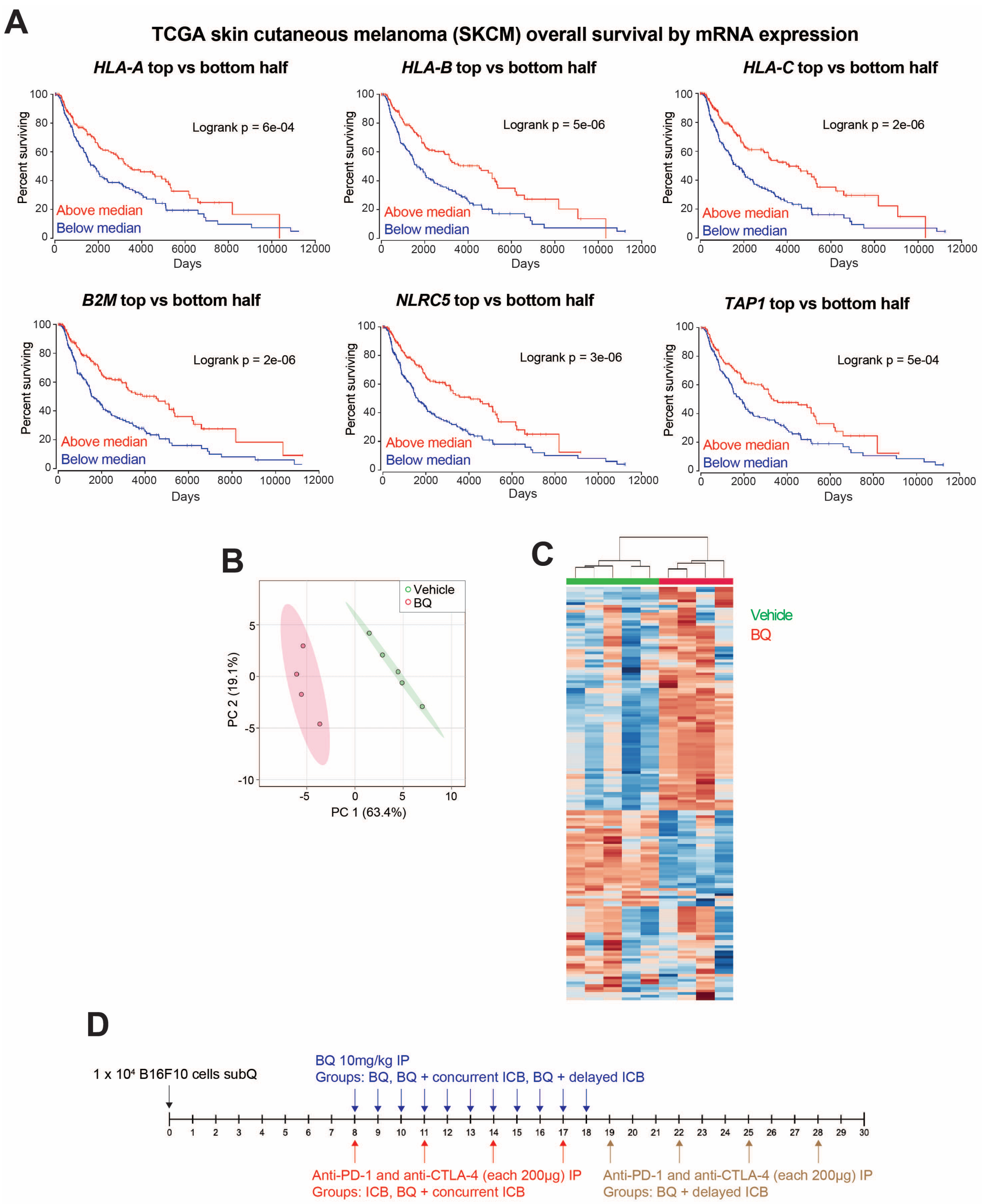

Figure S5: A) Overall survival in melanoma patients (SKCM) from the Cancer Genome Atlas (TCGA) with above (indicated in red) and below median (indicated in blue) mRNA expression of indicated genes. **B-C)** Principal component analysis (PCA) plot (B) and unsupervised hierarchical clustering (C) from metabolomics analysis of B16F10 tumors at necropsy (Fig 5C). **D)** Treatment regimen of mice from Fig 5E.
